# Monozygotic twins affected by SAPHO syndrome^☆^

**DOI:** 10.1016/j.abd.2024.03.008

**Published:** 2024-10-31

**Authors:** Ilaria Scandagli, Elia Rosi, Gianmarco Silvi, Matteo Ruggieri, Tommaso Amadori, Francesca Prignano

**Affiliations:** a Department of Health Sciences, Section of Dermatology, University of Florence, Florence, Italy; b Department of Radiology, Careggi University Hospital, Florence, Italy

Dear Editor,

Monozygotic twin boys, 15-year-old, born to non-consanguineous parents, presented with a 5-month history of severe and painful acneiform ulcerative lesions on their face, back and chest. Patient 1 reported sternal and intense lower back pain, while patient 2 had mild lower back pain. In addition, their mother was concerned about the rapid weight loss in both children. Their medical records revealed a one-year history of moderate acne and recurrent nodules in the armpits and groins. The family history for acne and joint disease was negative. Both twins had inflammatory nodules, comedones, pustules as well as painful hemorrhagic ulcers with adherent crusts on the back, chest and occipital region (Fig. 1). We observed multiple nodules and draining fistulas in both their inguinal and axillary areas. Based on clinical findings a preliminary diagnosis of Acne Fulminans (AF) in combination with Hidradenitis Suppurativa (HS) was made, and oral corticosteroid therapy (prednisone 0.7 mg/kg/day) in combination with topical clindamycin for inguinal and axillary lesions was started, to be applied once a day. After two weeks of treatment, the ulcerative lesions improved; therefore, prednisone was tapered by 5 mg every 7 days for each twin. Laboratory investigations showed a raised erythrocyte sedimentation rate and C-reactive protein in both patients. However, the complete blood cell count and differential, alkaline phosphate, calcium, phosphate, and antinuclear antibodies, rheumatoid factor, and anti-cyclic citrullinated peptide antibody were within range. Because of chest and back pain and inflammatory markers increase, we referred our patients to the Rheumatology and Radiology departments. Magnetic Resonance Imaging (MRI) of patient 1 showed altered signal intensities of the left sacroiliac joint and of the posterior arch of the fourth rib on the right, while for patient 2, MRI highlighted altered signal intensity at the left sacroiliac joint (Fig. 2). Evidence of sacroiliitis in both led to a diagnosis of Synovitis-Acne-Pustulosis-Hyperostosis-Osteitis Syndrome (SAPHO). Adalimumab, a medication targeting Tumor Necrosis Factor (TNF)-alpha, at a dose of 40 mg every 14 days, along with zolendronate and oral prednisone, was started. After six months, there was a marked improvement in both the acne and HS lesions (Fig. 3). Bone discomfort was no longer reported. Additionally, systemic inflammation markers level normalized and both twins exhibited weight gain.

**Figure 1:**
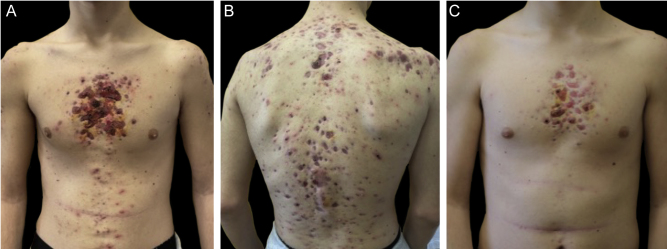
Upon clinical presentation, both patient 1 (A‒B) and patient 2 (C) exhibited ulcerative acneiform lesions on their chest, leading to a diagnosis of acne fulminans.

**Figure 2:**
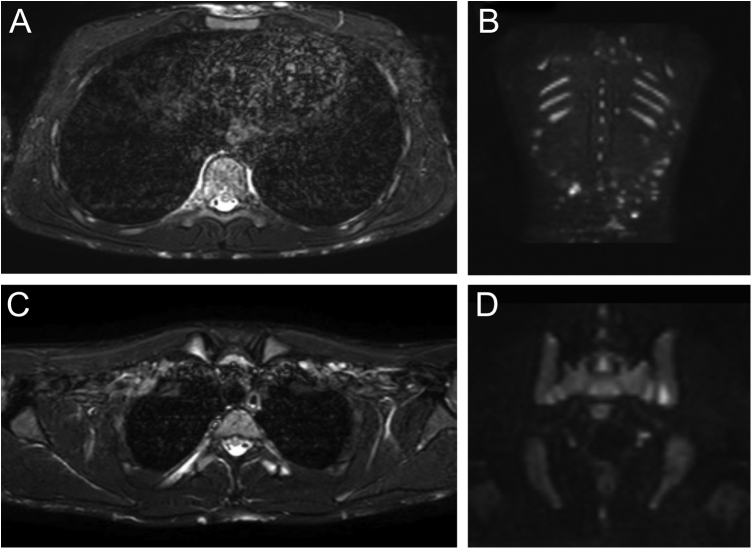
Patient 1: Axial Short Tau Inversion Recovery (STIR) MRI images (left) of the chest revealing nodular hyperintensity signals in the cutaneous-subcutaneous region affecting the anterior chest wall as well as the back (A); hyperintensity signal of the posterior arch of the fourth rib on the right due to marrow edema but no edema of the surrounding soft tissues (B). Coronal Diffusion-Weighted Whole-Body Imaging with Background Body Signal Suppression (DWIBS) images (right) demonstrating increased signal at the level of multiple subcutaneous alterations of the patient's back (C); focal hyperintensity signal at the left sacroiliac joint (D).

**Figure 3:**
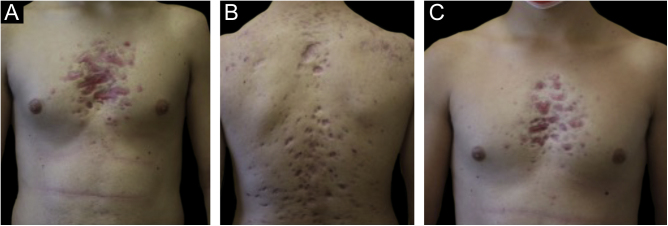
After six months, the patient 1 (A‒B) and the patient 2 (C) had atrophic scars on their chest and back.

Despite SAPHO remission in both patients, HS's chronic nature required a targeted treatment approach. As adalimumab is one of the few approved treatments for HS, it was decided to continue the long-term administration. Dermatological-rheumatological follow-up appointments are scheduled every six months.

SAPHO syndrome is a rare disorder, affecting 1 in 10,000 Caucasians.^1^ Although its pathogenesis is unknown, it’s recently been suggested to be auto-inflammatory. However, a combination of hereditary, infectious, and immunological factors might contribute to disease onset and development.^2^ The diagnosis of SAPHO syndrome is based on history, clinical, and findings supported by imaging investigations. Currently, there is no universal consensus among researchers concerning diagnostic criteria.^3^ Main differential diagnoses for SAPHO syndrome include infectious osteitis, osteomyelitis, malignancy, and other bone and joint disorders. The sternocostal and sternoclavicular joints and the costoclavicular ligament are the most involved. Radiological imaging may show hyperostotic bone changes, sclerotic lesions, osteolysis, periosteal reaction and osteoproliferative lesions involving enthesis.^4^ Acneiform and neutrophilic dermatoses are the key findings for SAPHO syndrome diagnosis. Notably, up to 60% of dermatological manifestations are palmoplantar pustulosis. 25% of patients, primarily males, experience moderate to severe nodulocystic acne. Other related conditions like HS, Sneddon-Wilkinson, Sweet syndrome and Pyoderma gangrenosum, are less common. Roughly 10% of SAPHO syndrome sufferers experience symptoms like fever and fatigue.^5^ Elevated inflammation markers, leukocytosis and mild anaemia might manifest in lab results. Treatment of SAPHO syndrome is necessary for symptom relief and prevention of further complications such as impairment of bone and joint function. Treatment strategies are often based on evidence from case reports and may vary depending on the disease manifestations in the patient; this is primarily because clinical trials have not been conducted because of its rarity. A nonsteroidal anti-inflammatory drug and short-term glucocorticoids provide initial osteoarticular relief. Methotrexate can treat peripheral arthritis in the absence of axial disease, while TNF inhibitors are preferred for severe enthesitis and axial disease. For patients presenting with both osteoarticular and skin manifestations in SAPHO syndrome, healthcare providers may consider diverse approaches, such as oral retinoids for palmoplantar pustulosis and acne. Viable options for challenging cases include bisphosphonate therapy, Interleukin (IL)-17 inhibition, IL-1 inhibition, IL-12/23 inhibition and Janus kinase inhibition.^6^ Our twin patients’ response to adalimumab matches existing literature documenting its efficacy for SAPHO syndrome. This report is unique due to the simultaneous and rare occurrence of AF and HS in monozygotic twins as part of the SAPHO syndrome.

The data that support the findings of this study are available from the corresponding author upon reasonable request.

## Financial support

None declared.

## Authors’ contributions

Ilaria Scandagli: Concepts, design, definition of intellectual content, manuscript preparation.

Elia Rosi: Definition of intellectual content, manuscript preparation, manuscript editing, Manuscript review.

Gianmarco Silvi: Literature search, manuscript review.

Matteo Ruggieri: Definition of intellectual content, literature search, manuscriptpreparation.

Tommaso Amadori: Manuscript preparation, manuscript editing, manuscript review.

Francesca Prignan: Concepts, definition of intellectual content, manuscript editing, manuscript review, guarantor.

## Conflicts of interest

None declared.
